# Long-term trends of phosphorus concentrations in an artificial lake: Socio-economic and climate drivers

**DOI:** 10.1371/journal.pone.0186917

**Published:** 2017-10-19

**Authors:** Yuliya Vystavna, Josef Hejzlar, Jiří Kopáček

**Affiliations:** Biology Centre CAS, Institute of Hydrobiology, České Budějovice, Czechia; University of Hyogo, JAPAN

## Abstract

European freshwater ecosystems have undergone significant human-induced and environmentally-driven variations in nutrient export from catchments throughout the past five decades, mainly in connection with changes in land-use, agricultural practice, waste water production and treatment, and climatic conditions. We analysed the relations among concentration of total phosphorus (TP) in the Slapy Reservoir (a middle reservoir of the Vltava River Cascade, Czechia), and socio-economic and climatic factors from 1963 to 2015. The study was based on a time series analysis, using conventional statistical tools, and the identification of breaking points, using a segmented regression. Results indicated clear long-term trends and seasonal patterns of TP, with annual average TP increasing up until 1991 and decreasing from 1992 to 2015. Trends in annual, winter and spring average TP concentrations reflected a shift in development of sewerage and sanitary infrastructure, agricultural application of fertilizers, and livestock production in the early 1990s that was associated with changes from the planned to the market economy. No trends were observed for average TP in autumn. The summer average TP has fluctuated with increased amplitude since 1991 in connection with recent climate warming, changes in thermal stratification stability, increased water flow irregularities, and short-circuiting of TP-rich inflow during high flow events. The climate-change-induced processes confound the generally declining trend in lake-water TP concentration and can result in eutrophication despite decreased phosphorus loads from the catchment. Our findings indicate the need of further reduction of phosphorus sources to meet ecological quality standards of the EU Water Framework Directive because the climate change may lead to a greater susceptibility of the aquatic ecosystem to the supply of nutrients.

## Introduction

The present trends in the quality of aquatic ecosystems generally reflect those of socio-economic and climate conditions [1–3]. The relationship between water quality and human-induced environmental alterations has become more pronounced during past five decades [1, 4], and has manifested through an elevated nutrient loading into water bodies and deterioration of surface waters [5–7]. Agricultural activities (application of fertilizers, livestock production, soil cultivation), population growth, and infrastructure development (connection to sewerage and wastewater treatment facilities, increasing treatment efficiency of wastewaters) have been important socio-economic factors influencing nutrient balance in numerous catchments for more than a century [8–11] and can be responsible for the development of eutrophication [1, 12]. Additionally, recent climate change has become an important factor that affects the nutrient dynamics of catchments [13, 14] by changing surface water temperatures [15–17], frequency and amount of precipitation [14, 18, 19], seasonality and magnitude of water flows [12, 16], and the duration of thermal stratification of lakes [5, 17, 20, 21].

Changes in nutrient inputs have cascading effects across aquatic food webs through various direct and indirect interactions [22]. Despite the recent increase in attempts to regulate the production and application rate of nutrients (nitrogen, N; and phosphorus, P) in economic sectors, i.e. Nitrate Directive (ND 91/676/EEC), Urban Wastewater Treatment Directive (UWTD 91/271/EEC), and European Water Framework Directive (WFD 2000/60/EC), problems of anthropogenic eutrophication still represent an important issue worldwide [8, 22–26]. The high risk of eutrophication is especially prominent in catchments where runoff is intensively regulated by dams, and those affected by human activities like urbanisation or agriculture [12].

In many central European catchments, P concentrations increased from the 1960s to 1980s and then declined reflecting the changes in human activities and regional socio-economic conditions (e.g., [27–31]). However, several studies indicate that in-lake P concentrations and trophic conditions reflect not only the P-load changes but also are influenced by processes in the lake ecosystem that can either strengthen [32, 33] or weaken [34, 35] the decreasing P-load trends in the inflow. Climate change is a factor that can act both at the level of P export from the catchment and at the level of processes in the lake ecosystem. For example, the expected variations in frequency and intensity of precipitation might increase P export from the catchment diffuse sources [31, 36, 37]; on the other hand, implementation of anti-erosion protection measures can reduce P loads from agricultural areas [13, 29, 38]. But, as a whole, it can be concluded that the relationships between trends in P concentrations in surface waters and socio-economic and climate variables still have been poorly evaluated at the long-term scale, despite the high importance of P for the eutrophication of freshwaters [4, 39, 40].

The aim of our study was to analyse long-term variations in total phosphorus (TP) concentration in the Slapy Reservoir (SL), i.e. a middle reservoir in the chain of reservoir at the Vltava River, Czechia, and socio-economic and climatic factors from 1963 to 2015. Similar long-term datasets are rare, and hence, of great importance due to the possibility to test various hypotheses linking water quality and plankton ecology to the climate-driven and human-induced changes [41–43] that differs from other studies on short term P data in lakes [32]. In our study, we tested the hypothesis that long-term TP concentrations in SL reflect the key catchment activities causing P loss into the river network and also the recent climate warming by influencing the seasonality of P availability and in the aquatic ecosystem.

## Materials and methods

### Study site description

**Fig 1:**
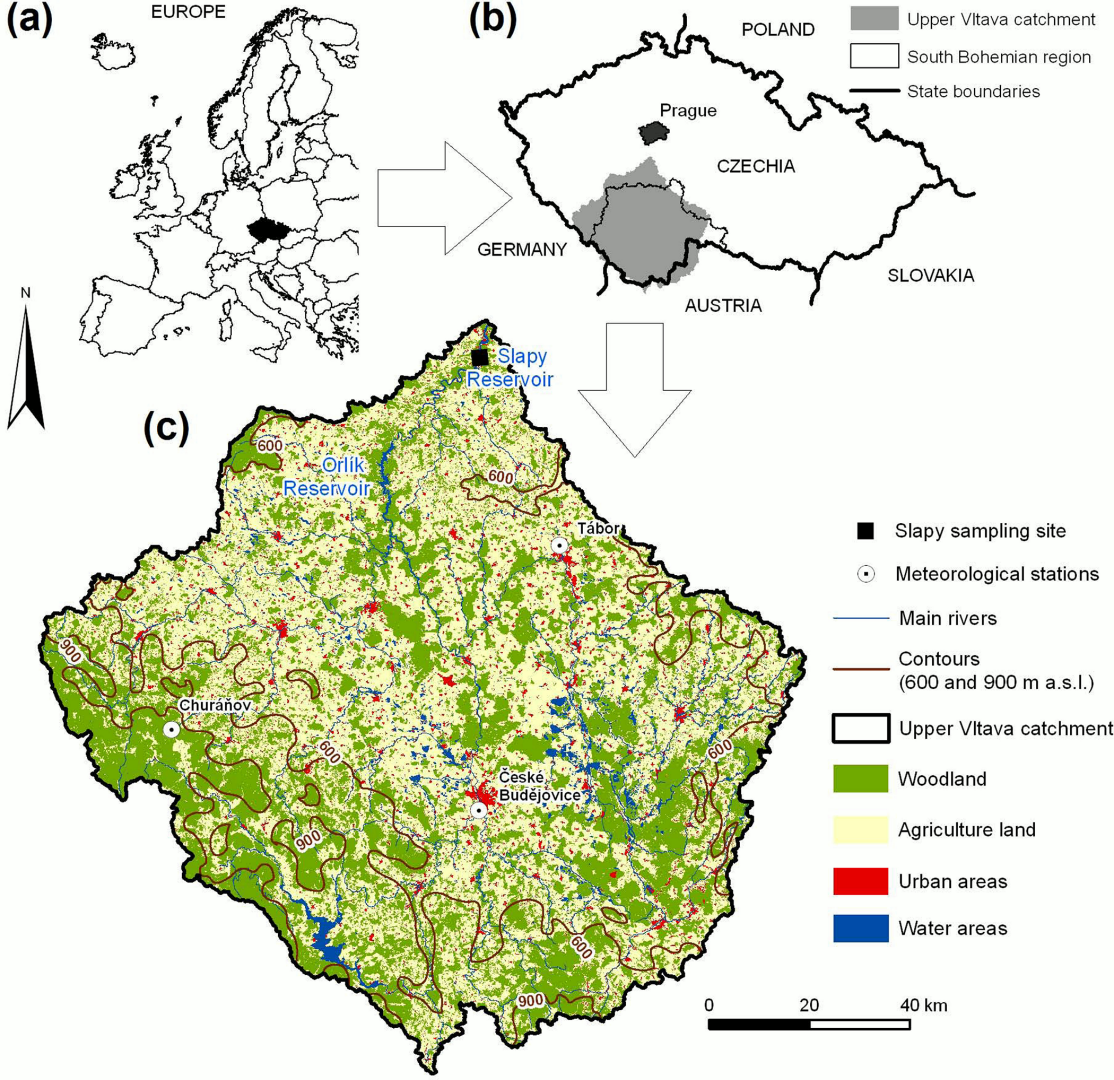
Location (a,b) and characteristics (c) of the Slapy Reservoir catchment.

The upper Vltava River catchment (~13,000 km^2^) is typical for the Central European region in terms of the socio-economic development and environmental conditions [10, 11, 44]. The Vltava River is regulated by a cascade of reservoirs that are primarily used for hydropower production, flood protection and water supply [45]. The catchment of SL (12,968 km^2^, elevation of 271−1,378 m a.s.l.) covers the entire upper Vltava basin and stretches from the Bohemian Forest mountain range between Czechia and Austria/Germany to the Slapy dam, built ~40 km south (and upstream) of Prague and finished in 1955 [46] (Fig 1). It is coincident in size with the administrative South Bohemia region, with available statistical data on agricultural and human activities. The Orlík Reservoir (altitude of 354 m a.s.l., surface area of 27.3 km^2^, volume of 0.717 km^3^, dam ~70 km south of Prague) was built upstream and close to SL and put in full operation in 1963 [46]. SL is a 42 km long, narrow (mean width is ca. 310 m) and deep (maximum depth is 53.5 m and average depth is 23.2 m) fjord-like water body. At the operational level of 270.6 m a.s.l., its volume is 0.27 km^3^ and its surface area is 11.6 km^2^. The average (1963–2015) water flow (Q) at the dam was 90 m^3^ s^-1^. The outflow from SL is commonly via the intakes of the hydropower station at a depth of ca. 40 m. The inflow into SL is largely formed by the discharges from the low outlets of the Orlík Reservoir, which modifies the seasonal temperature pattern: the inflow water is relatively colder during the spring and summer while it is warmer in the autumn and winter compared to the water temperature (T_w_) in the Vltava River upstream from the Orlík Reservoir [45]. Therefore, during the vegetation period (from late spring, when the air temperature (T_a_) increases above ca. 10°C), a cold inflow water passes SL through the hypolimnion, with restricted mixing to its epilimnion. In contrast, the entire SL water column is mixed from autumn to spring and the lake is usually ice-free during the winter [45, 47]. SL can therefore be considered as a monomictic water body despite its geographic location.

The locality of SL has a climate typical of the temperate zone, with annual average T_a_ of 8.2°C and annual precipitation of 582 mm during 1963–2015 (the Tábor meteorological station; data by the Czech Hydrometeorological Institute (CHMI)). Agricultural land, forests (mostly plantations of Norway spruce, *Picea abies*), surface waters, and urban areas cover 52%, 42%, 3%, and 3% of the catchment, respectively. Point source pollution is primarily due to wastewater discharges from municipalities and wastewater treatment plants (WWTP) and diffuse pollution is mainly from agricultural land [10, 11].

### Socio-economic indicators

The socio-economic development of the SL catchment shown changes typical for central and eastern European countries, i.e. shifts from the market to the planned economy in the 1950s and a return to the market economy in the 1990s [10, 44]. Factors controlling nutrient exports from agricultural lands in this large heterogeneous catchment have been documented since the 1900s, namely factors responsible for trends in N and sulphate concentrations in SL [10, 11, 44]. Socio-economic indicators were selected in order to represent the national economic development, regional agricultural activity, population growth and infrastructure development [10, 11, 26] (Table 1).

**Table 1:** Socio-economic and environmental indicators.

Indicator	Description	Unit	Time period	Source
*Socio-economic indicators*				
**Gross domestic product (GDP)**	Characteristics of the general trend of national economic development of Czechia in a real price value	USD per person	Annual, from 1970 to 2015	OECD data base (https://data.oecd.org/gdp/gross-domestic-product-gdp.htm)
**Organic fertilizers (Fo)**	Application of phosphorus through organic fertilizers in the South Bohemian region per area of agriculture land	kg P per hectare	Annual, from 1963 to 2015	Czech Statistical Office (https://www.czso.cz)
**Mineral fertilizers (Fm)**	Application of mineral phosphorus fertilizers in the South Bohemian region per area of agriculture land	kg P per hectare	Annual, from 1963 to 2015	Czech Statistical Office (https://www.czso.cz)
**Livestock density on agricultural land (LS)**	Characterized by animal units (AU = 500 kg of live weight; i.e., one cow or horse = 1 AU, one pig = 0.2 AU, one sheep or goat = 0.15 AU and one poultry = 0.004 AU) in the South Bohemian region	AU per hectare	Annual, from 1963 to 2015	Czech Statistical Office (https://www.czso.cz)
**Population (PO)**	Population of the South Bohemian region	Inhabitants	Annual, from 1963 to 2015	Czech Statistical Office (https://www.czso.cz)
**Connection to sewerage (X**_**S)**_	Share of the population connected to sewerage in the SL catchment	%	Annual, from 1963 to 2015	Czech Statistical Office (https://www.czso.cz)
**Connection to WWTP (X**_**W**_**)**	Share of the population connected to wastewater treatment facilities in the SL catchment	%	Annual, from 1963 to 2015	Czech Statistical Office (https://www.czso.cz)
**Phosphorus load to surface waters via wastewater (P**_**load**_**)**	Determination based on specific P load to wastewater from the population, share of the population connected to sewerage systems and sewage treatment facilities, type of wastewater treatment technologies (mechanical, biological, chemical), and evidence of P discharges from WWTPs in the SL catchment	Mg P per year	Annual, from 1963 to 2015	Calculated according to [11]
**Specific P load to wastewater from population (P**_**spec**_**)**	Human waste contribution to P load in municipal wastewater in the SL catchment	g P per person per day	Annual, from 1963 to 2015	Measured in sewerage of representative municipalities in the SL catchment
*Environmental indicators*				
**Total phosphorus (TP)**	Samples taken from a depth of 0.5 m (epilimnion) in SL	μg L^-1^	Three-week intervals from 1963 to 2015	Measured
**Chlorophyll-*a* (Chla)**	Integrated samples taken from depths 0–3 m in surface layer (epilimnion) in SL	μg L^-1^	Three-week intervals in 1963–1969 and 1979–2015	Measured
**Water temperature (T**_**w**_**)**	Water temperature measured at the 0.5 m depth (epilimnion) in SL	°C	Three-week intervals from 1963 to 2015	Measured
**Air temperature (T**_**a**_**)**	Data from meteorological stations in the SL catchment, i.e., Tábor, České Budějovice and Churáňov	°C	Daily average, from 1963 to 2015	Czech Hydrometeorological Institute (http://portal.chmi.cz/)
**Water flow (Q)**	daily data on the inflow into SL	m^3 ^s^-1^	Daily average, from 1963 to 2015	Vltava River Board, (Povodí Vltavy, státní podnik: http://voda.gov.cz/)

### Environmental indicators

The SL water samples were taken in three-week intervals at the Živohošť Bridge (49.7657N, 14.4134E; 8.8 km upstream from the dam) ca. 0.5 m below the surface from 1963 to 2015. Samples were immediately filtered through a 200-μm polyamide sieve to remove zooplankton and/or other coarse particles, transported to the laboratory and analyzed for TP within a day after sampling. The TP was determined using perchloric acid digestion followed by the molybdate method according to Popovský [48] in 1963–1991, then by the semi-micro modification of this method [49]. In both modifications, samples were concentrated by evaporation (with diluted perchloric acid at ~100°C) prior to digestion in order to obtain a detection limit of ~1 μg l^–1^ P. Both modifications of the method provided almost identical results [49]. During years 2004 and 2006, TP concentration was analysed in samples taken at the same site from the depth of 40 m by a Friedinger sampler. The chlorophyll-*a* (Chla) was determined in unfiltered integrated samples from depths of 0 to 3 m by spectrophotometric methods after aceton extraction according to Strickland and Parsons [50] or Lorenzen [51] in periods between 1963–1969 and 1979–2015, respectively. T_w_ was measured at the depth of 0.5 m during the water sampling (Table 1). The daily Q data were originated from the operating records at the SL dam and were obtained from the Vltava River Board, state enterprise, Prague (Table 1). Daily average T_a_ data from three long-term operated meteorological stations in the SL catchment, namely Tábor (49.4362N, 14.6581E; altitude of 459 m a.s.l.; World Meteorological Organization (WMO) ID 11582), České Budějovice (48.9519N, 14.4687E; altitude of 395 m a.s.l.; WMO ID 11546) and Churáňov (49.0682N, 13.6151E; altitude of 1,120 m a.s.l.; WMO ID 11457) (Fig 1) were obtained from the CHMI (Table 1).

### Data analysis

The data on T_a_, Q, and T_w_ were recalculated into monthly, seasonal and annual average values, applying standard arithmetic average function. Seasons were defined as winter (December–February), spring (March–May), summer (June–August) and autumn (September–November). The log-normality of the data was tested using the Kolmogorov–Smirnov test. Trends for the seasonal data were identified using the seasonal Kendall test, which is a non-parametric technique for detecting monotonic trends [52]. The seasonal Kendall test is particularly useful for monitoring data because the test is not influenced by missing values and is insensitive to outliers. The relationships between variables were evaluated using linear regressions and Pearson correlations. Breaking points in the long-term data were identified by applying segmented regression (SegReg program, developed by Institute for Land Reclamation and Improvement, Netherlands, http://www.waterlog.info/segreg.htm). The segmented regression has been used in water quality studies to detect breaking points in different datasets [53–55]. This regression is based on the use of a linear predictor represented by two or more straight lines connected by an unknown breaking point [54]. Time was used as an independent variable and TP concentration, T_a_, T_w,_ and Q were dependent variables. All statistical analyses were performed at a 95% confidence level (p < 0.05) at least.

## Results

### Long-term and seasonal variations of total phosphorus concentration

**Fig 2:**
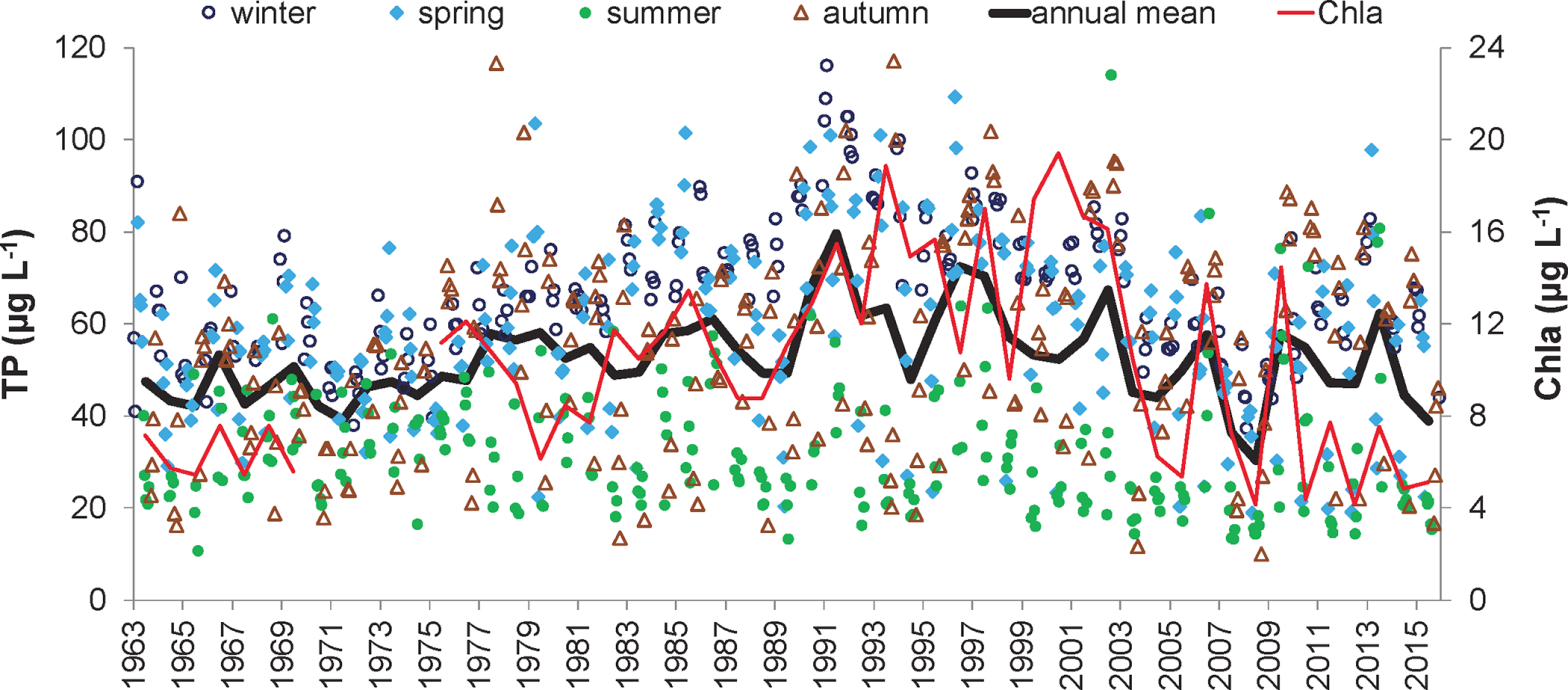
Time-series of total phosphorus (TP) and chlorophyll-a (Chla) concentrations in the Slapy Reservoir during 1963–2015. The black line is the annual average TP concentration; points are individually measured TP concentrations with indicated sampling seasons; the red line is the annual average concentration of Chla.

Individually measured TP concentrations varied greatly (between 10 and 118 μg L^–1^) in SL during the 53-year period of observation (Fig 2). Annual average TP concentrations ranged during the study period from 31 to 77 μg L^-1^ and were similar to their annual median values (35–83 μg L^-1^). The Kendall test indicated no statistically significant time trends in the annual and seasonal average TP concentrations for the whole (1963–2015) study period. However, the segmented regression indicated a breaking point in annual average TP concentrations in 1992. The Kendall test confirmed two significant trends (p < 0.001) of annual average TP concentrations; the first had a slope of 0.55 mg L^-1^ yr^-1^ in 1963–1991 and the second had a slope of -0.85 mg L^-1^ yr^-1^ in 1992–2015. The epilimnetic concentrations of Chla (Fig 2) closely correlated with TP concentrations (Pearson correlation, *r* = 0.59, p < 0.01).

**Fig 3:**
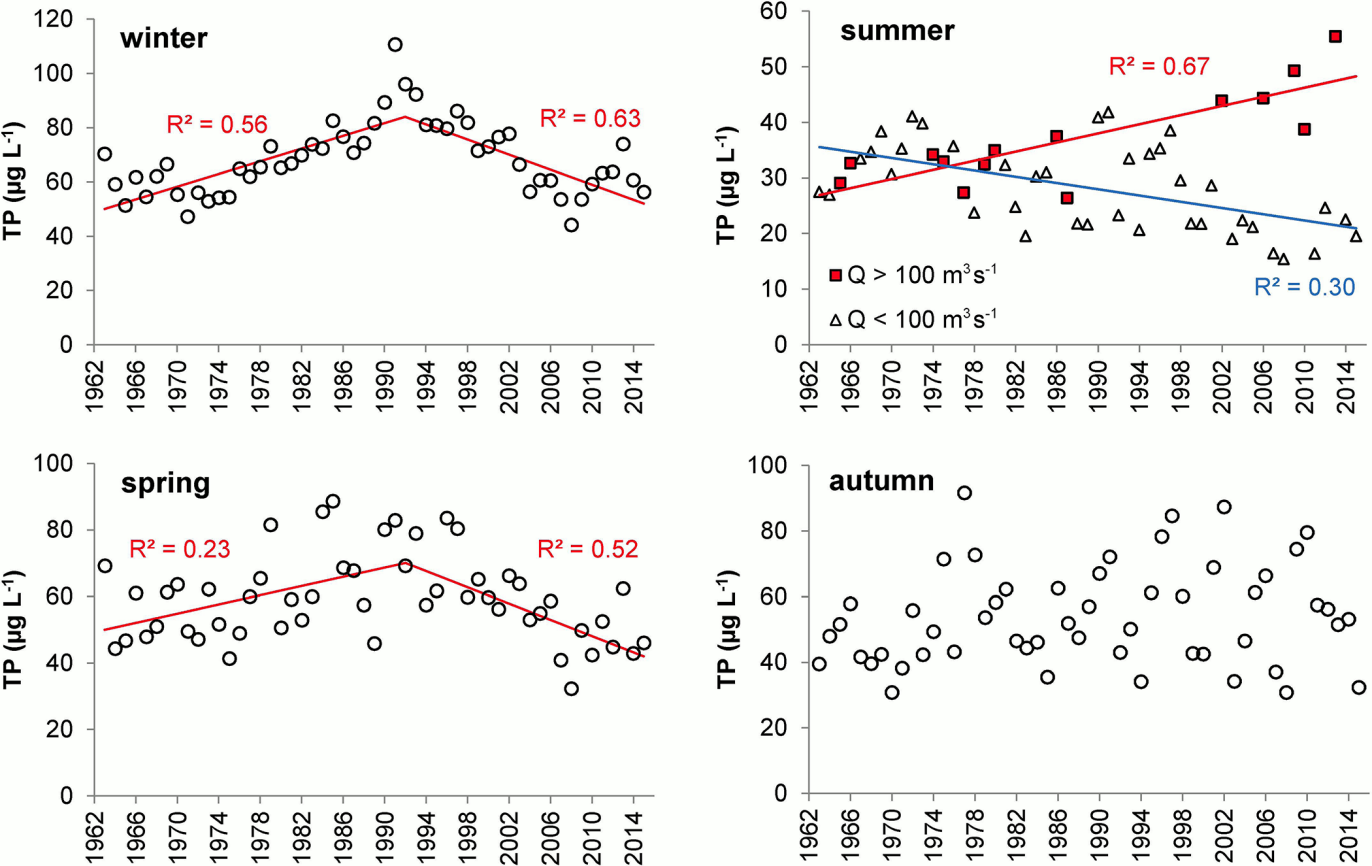
Seasonal average total phosphorus concentrations (TP) in the Slapy Reservoir during 1963–2015. Lines indicate significant linear trends (p < 0.05).

The seasonal average TP concentrations exhibited large differences, with the lowest values in summer and the highest ones in winter (Fig 3). Moreover, the segmented regression revealed two significant trends in the winter and spring average TP concentrations, with a breaking point in 1992 when the increasing trend (1963–1991) reversed to a decreasing trend (1992–2015) (Fig 3). Summer and autumn average TP concentrations were more dispersed, with no significant trends or breaking points. However, within the scatter of summer TP concentrations, two opposite significant trends were identified when the TP values were divided into two groups according to the average summer Q, i.e. the trend was positive for Q > 100 m^3^ s^-1^, but negative for Q < 100 m^3^ s^-1^ (Fig 3).

**Fig 4:**
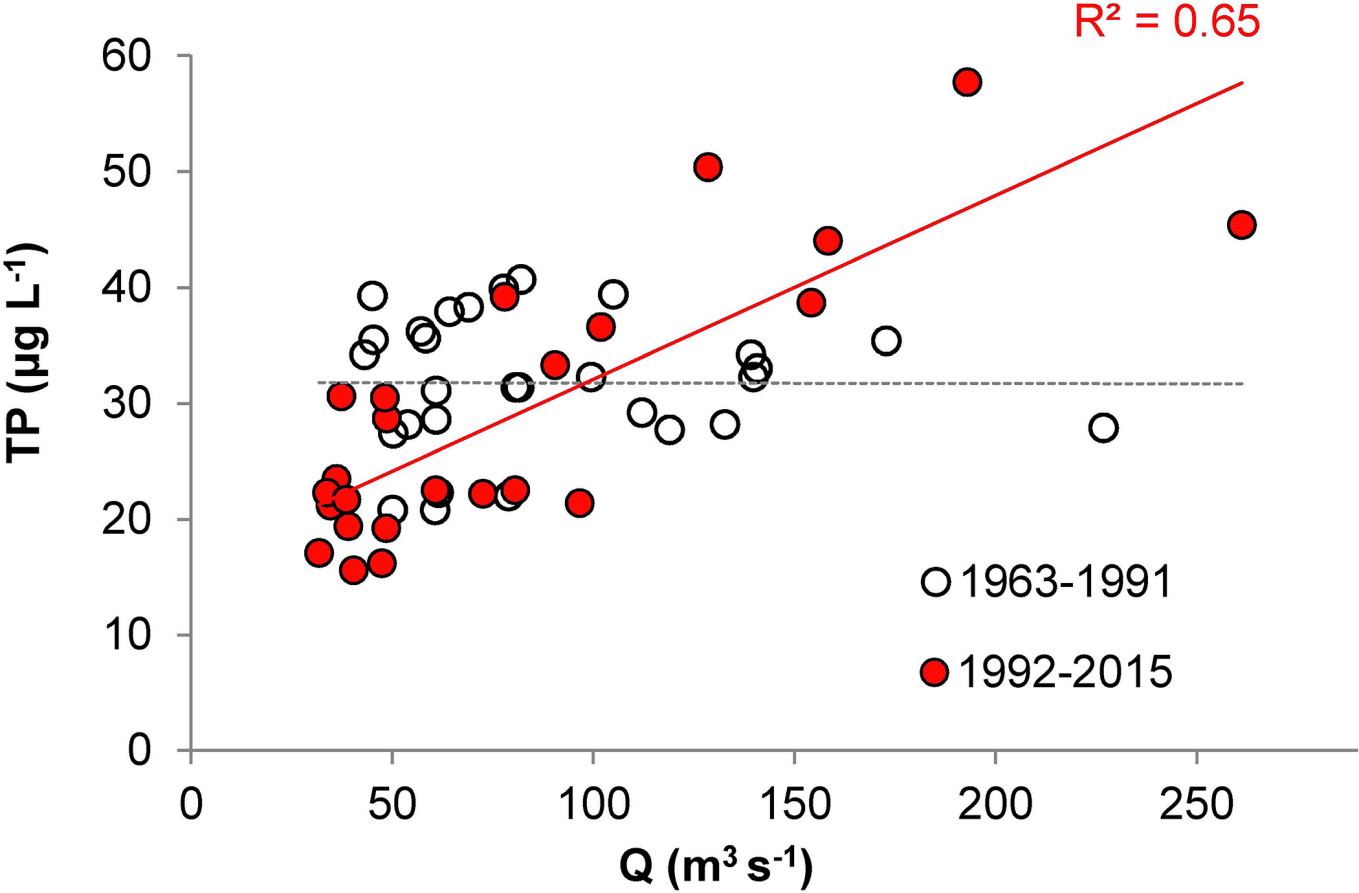
The relationships between summer average water flow (Q) and total phosphorus concentration (TP) in the Slapy Reservoir during 1963–1991 and 1992–2015. The red line is a significant (p < 0.001) trend in 1992–2015; the broken gray line is an insignificant (p > 0.05) trend in 1963–1991.

Regression analysis of summer average TP and Q values revealed that the TP values were independent of Q from 1963 to 1991, while positively correlated with Q from 1992 to 2015 (Fig 4).

**Fig 5:**
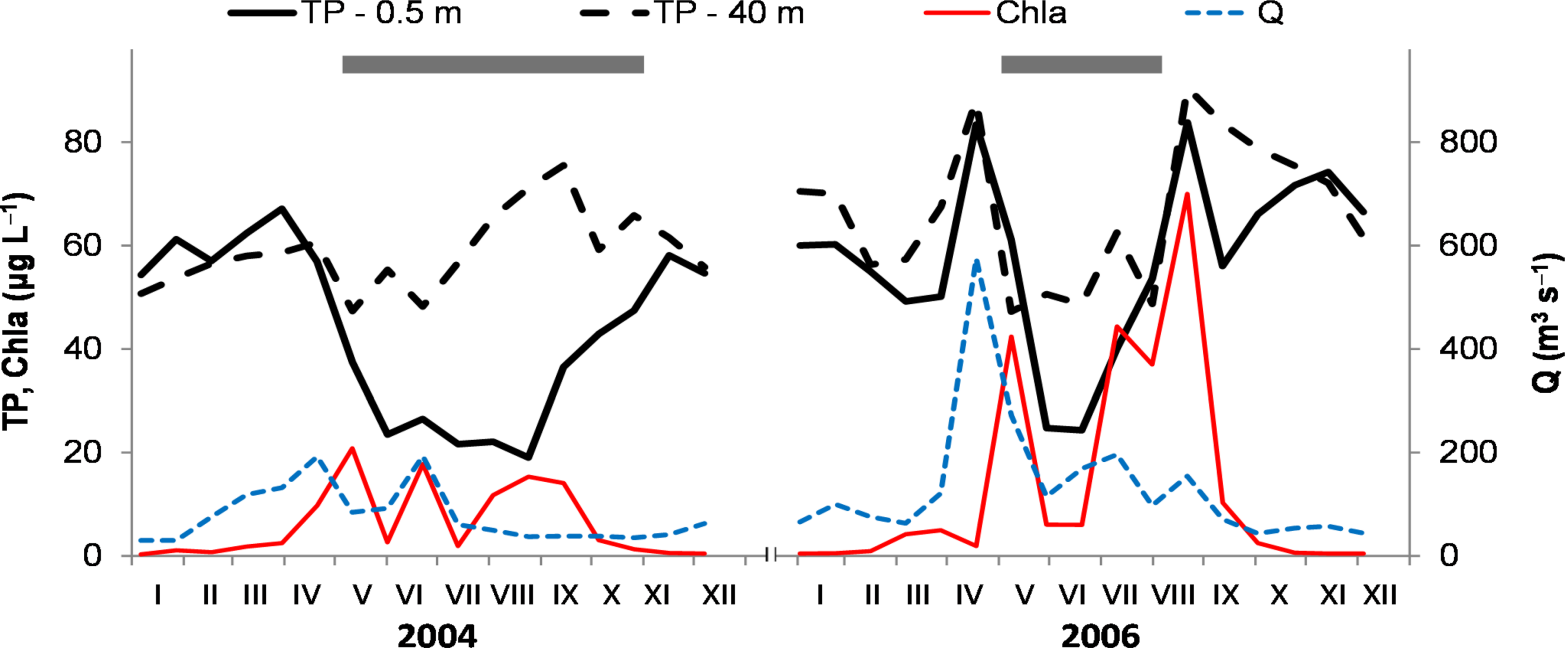
Total phosphorus (TP) and chlorophyll-*a* (Chla) in the Slapy Reservoir during two years with different water flow. TP-0.5 m and TP-40 m are TP concentrations in the epilimnion and the hypolimnion, respectively; Chla is in the epilimnion (0–3 m); Q is water flow; grey horizontal bars denote periods with a thermally stratified water column.

The measurements of TP concentrations in the SL water column profile in 2004 and 2006 indicated gradually increasing TP in the hypolimnion during the May–October period of thermal stratification and showed significant increases in the epilimnetic TP after high Q events in 2006, followed by an elevated Chla in the epilimnion (Fig 5).

### Socio-economic indicators

**Fig 6:**
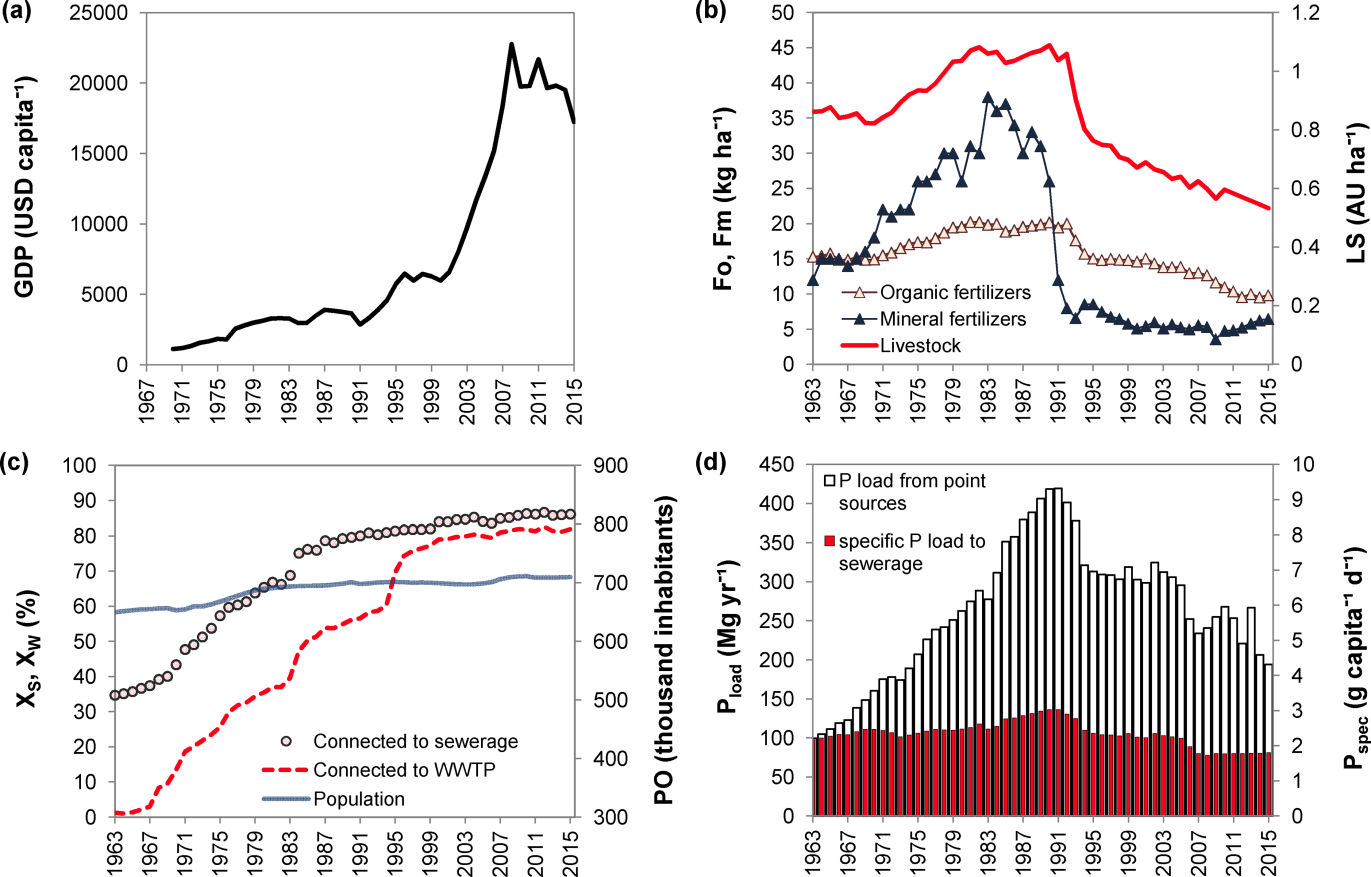
Socio-economic indicators of the Slapy catchment in 1963–2015. (a) Gross domestic product (GDP); (b) agricultural activity: application of organic (Fo) and mineral (Fm) fertilizers, livestock (LS); (c) infrastructure development: number of population (PO), population connected to sewerage (X_s_) and connected to WWTP (X_w_); and (d) specific per-capita P contribution to wastewater (P_spec_) and P loading from sanitary systems to surface waters (P_load_).

The socio-economic indicators have changed considerably during the last half-century, showing the highest changes between 1990 and 2006 (see, e.g. gross domestic product, GDP, in Fig 6A). Agricultural production markedly intensified during 1963–1990 when the application of P in mineral and organic fertilizers increased from 12 to 37 kg ha^-1^ and from 15 to 20 kg ha^-1^, respectively, and the livestock numbers (animal units, AU) increased from 0.8 to 1.1 AU ha^-1^. In the next period, during 1991–2015, the application of P in mineral and organic fertilizers decreased to 6 and 10 kg ha^-1^, respectively, and livestock numbers dropped to 0.5 AU ha^-1^, reaching a lower level than in 1963 (Fig 6B). The population in the SL catchment continued growing slightly during the whole study period while the sanitary infrastructure rapidly developed and the proportion of the population connected to sewerage increased faster than the proportion of wastewater treated in WWTPs (Fig 6C). The proportion of population connected to sewerage and sewerage with WWTPs reached 84% and 81%, respectively, by 2015 (Fig 6B). The specific per-capita production of P to wastewater (P_spec_) increased from 2.0 to 2.9 g person^–1^ day^–1^ and the P loads to surface waters increased via wastewater from ~100 to 420 Mg year^-1^ in 1963–1990 while these indicators decreased to 1.8 g person^–1^ day^–1^ and to 200 Mg year^-1^, respectively, until 2015 (Fig 6D).

The Pearson correlation indicated positive relationships between the annual average TP concentrations and P load to surface water via wastewater, application of organic fertilizers and livestock while there was a negative relationship between the TP and gross domestic product during 1963–2015 (Table 2). For the partial periods, however, the situation was different. In 1963–1990, the TP concentrations were positively correlated with the gross domestic product, population connected to WWTP (X_W_) and sewerage (X_S_), while these relationships became negative in 1991–2015 (Table 2).

**Table 2:** Pearson correlation between the annual average concentrations of total phosphorus (TP) in the Slapy Reservoir and socio-economic indicators its catchment during different time periods^a^.

TP in time period	Socio-economic indicators							
	P_load_	P_spec_	GDP	X_W_	X_S_	Fo	Fm	LS
**1963–2015**	0.53**	0.23	(-)0.27*	0.12	0.22	0.39**	0.11	0.32*
**1963–1990**	0.64**	0.56**	0.64**	0.64**	0.65**	0.62**	0.54**	0.63**
**1991–2015**	0.61**	0.65**	(-)0.59**	(-)0.50**	(-)0.61**	0.56**	0.40*	0.57**

^a^ numbers are *r*, the Pearson correlation criteria; negative sign (-) indicates negative correlation; asterisks indicate significance

*, p < 0.05

**, p < 0.01; the significant values are in the bold. For abbreviations see Table 1.

### Environmental indicators

**Fig 7:**
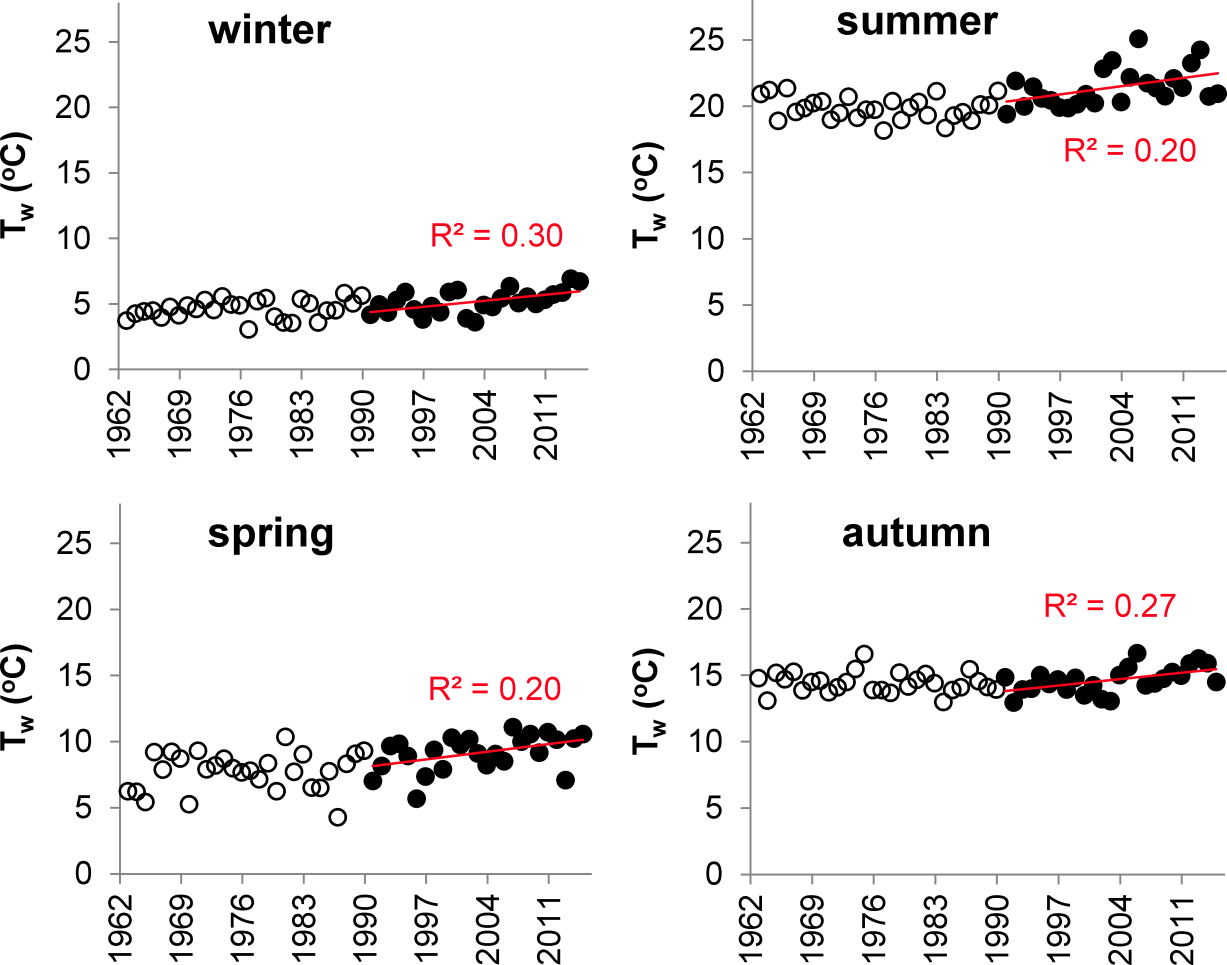
Long-term trends in the seasonal average water temperature (T_w_) in the Slapy Reservoir. Open and black points correspond to the years 1963–1990 and 1991–2015, respectively; regression lines indicate significant linear trends (p < 0.05) during 1991–2015.

The Kendall test applied to seasonal average data indicated that T_w_ in SL increased with a slope of 0.03°C yr^-1^ (p < 0.05) during the last 53 years. The segmented regression revealed a breaking point in 1987 for the annual average T_w_ data. For the seasonal average T_w_, significant increasing trends were indicated by Pearson correlation for each season in the period 1991–2015 (Fig 7).

**Fig 8:**
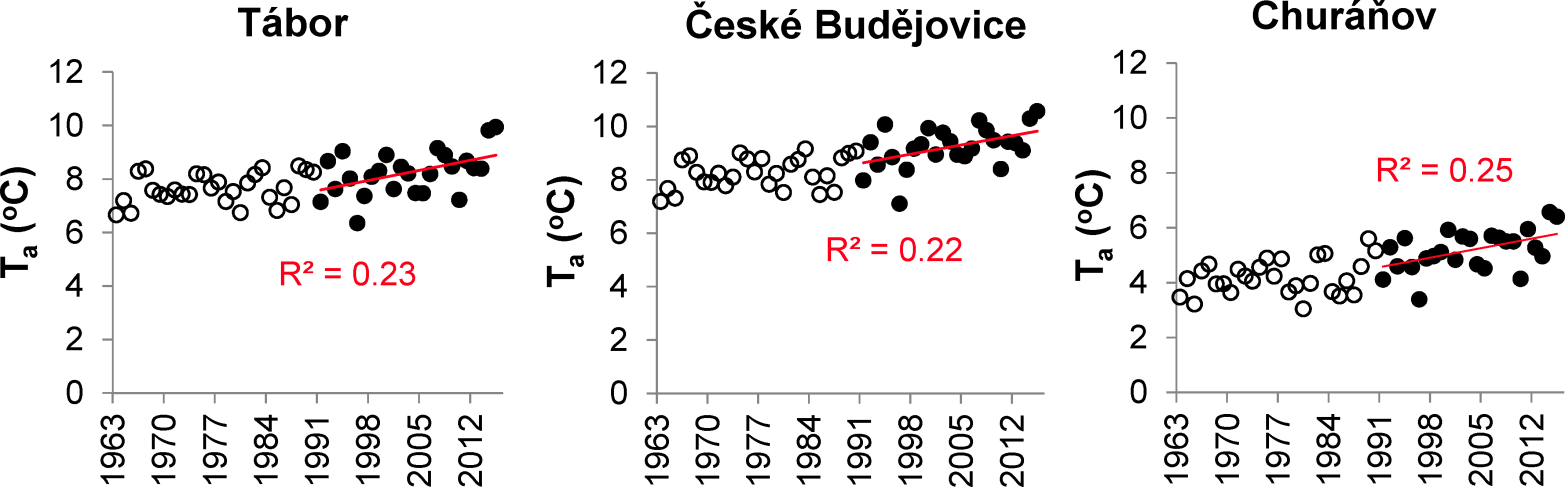
The annual air temperature (T_a_) measured at meteorological stations in South Bohemia (1963–2015). Open and black points correspond to the years 1963–1990 and 1991–2015, respectively; regression lines indicate significant linear trends (p < 0.05) during 1991–2015.

Similar to T_w_, T_a_ as measured at three stations in the SL catchment exhibited a significant increasing trend in 1991–2015 (Fig 8).

**Fig 9:**
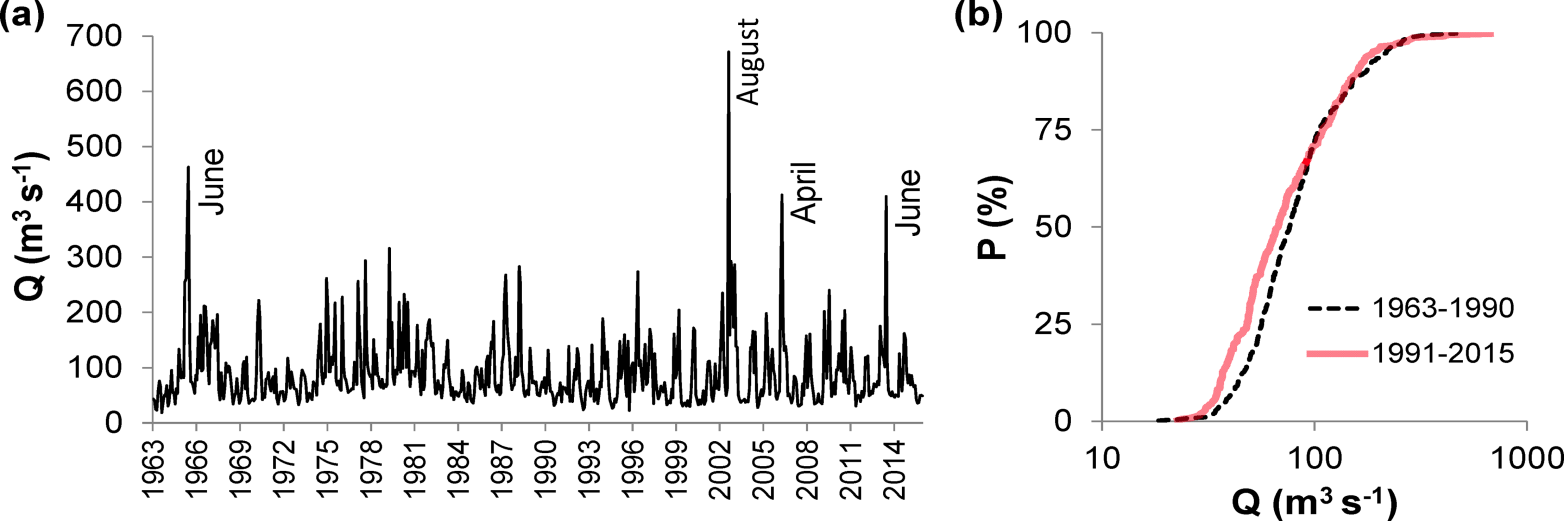
Monthly average water flow (Q) in the Slapy Reservoir. (a) Time-series of values during 1963–2015 (months with episodic high Q events are indicated) and (b) cumulative relative frequency distribution plots in 1963–1990 and 1991–2015.

The Kendall test and segmented regression indicated no statistically significant trends or breaking points for annual or seasonal data on Q. It was, however, observed that the distributions of monthly average Q were significantly different (p < 0.001 according to the Kolmogorov-Smirnov test) in the periods of 1963–1990 and 1991–2015 (Fig 9A), with higher frequencies of less-than-median Q but more frequent extreme Q in the latter period (Fig 9B). The extremely high Q events in the period of 1991–2015 occurred mainly in spring and summer (Fig 9A).

## Discussion

The results show significant relationships between TP concentrations in SL and socio-economic and climatic factors in its catchment during the period 1963–2015, hence confirming that our working hypothesis is valid. We found that trends in winter and spring TP concentrations in SL were related to socio-economic changes in the South Bohemian region. Significant positive relationships between TP concentrations *vs*. P loads from sanitary systems, application of mineral and organic fertilizers, and livestock production indicated that these drivers had a strong impact on TP variation in SL. The increasing and decreasing trends of winter and spring average TP concentrations during 1963–1991 and 1992–2015, respectively, (Fig 3) corresponded well to two socio-economic stages in Czechia, i.e. the development of agriculture and infrastructure (sewerage and sewage treatment facilities) in the years 1963–1990, followed by the reduction of agricultural activity, intensive development of wastewater treatment infrastructure, and regulation of consumption and application of P-containing products in agriculture and households between 1991 and 2015 (Fig 6B–6D).

The economic development in Czechia in 1963–1990 was based on the planned economy with the intensification of agricultural production [11, 56], increasing use of fertilizers, building of sanitary infrastructure and use of phosphate detergents and was an important driver of P loads in the SL catchment. This was confirmed by a simultaneous growth of TP concentrations, gross domestic product and P loads from agriculture and wastewater (Fig 6A, 6B and 6D), as well as by the positive correlation between these variables during this period (Table 2). The period of 1991–2015 was characterized by a simultaneous decrease in P loads from agriculture and untreated sewage (Fig 6B and 6C). This reduction occurred despite the continued rapid growth of the Czech gross domestic product (Fig 6A) and was associated with concurrent influences of various socio-economic factors. One of them was a temporary recession and restructuring of the economy [11]. Agricultural production rapidly decreased, mainly because of the rising prices of agricultural inputs (labour, fertilizers, techniques, etc.) and the absence of agricultural subsidies. A particularly marked decline was observed for the numbers of cattle and pigs, which resulted in the decrease of manure and slurry production [11]. The other important factor was a transformation in environment-related legislation that has been partly based on the ‘polluter-pays’ principle [57]. In 1991–2015, important environmental legislation changes included: (i) regulations of wastewater treatment efficiency and limits for P concentration in the treated wastewater, which were gradually set for all sizes of WWTPs with fees for the amount of discharged P being introduced for significant polluters (> 3 Mg of P per year) since 2005 [58]; (ii) the P load in wastewater originating from detergents was moderately reduced due to a voluntary agreement of the Czech Ministry of Environment with the producers of detergents in 1995, and eventually diminished by a legislative ban of phosphate in detergents for retail in 2006 [56, 58]; (iii) an agri-environment scheme that was implemented by the Czech Ministry of Agriculture after the accession of Czechia to the EU in 2004, which reduced losses of P from agricultural areas to surface waters from soil erosion and application of organic fertilizers [59].

A similar situation was observed in other European countries [60]. The legislative regulations for wastewater treatment and the related development of sewage treatment infrastructure in the late 1990s directly lead to a reduction of P discharge in many European catchments [61, 62]. In the agricultural sector, organic and mineral fertilizers were applied in excess in all European countries until 1980 to increase crop yields, which resulted in an intensive accumulation rate of P in the soil up to 15 kg ha^-1^ per year [62, 63]. Since the 1980s, inputs of fertilizers decreased gradually in Western Europe and dropped sharply in Eastern Europe in the 1990s [62]. However, despite reduced P emissions into the environment, soil and sediments may still release the previously accumulated P liberated during organic matter decomposition or under reducing conditions, but the importance of these sources has been decreasing [9, 62, 63].

The relationships between socio-economic trends in P sources and TP concentrations in SL were apparent in winter and spring, but not in summer and autumn (Fig 3). This can be explained by a stronger influence of additional drivers, e.g. T_w_ and Q, on TP variations during summer and autumn. T_w_ controls the length of the vegetation period, which starts in SL generally in April and continues till September, depending on the stability of thermal stratification [45]. The vegetation period in SL is characterized by the intensification of primary production and P uptake in aquatic food webs, i.e. phytoplankton, periphyton, macrophytes, zooplankton and fish [12]. The growth of their biomass is more pronounced during increased T_w_ and prolonged vegetation periods [64] and results in more depleted TP concentrations in the epilimnion.

Our long-term observation revealed that the most significant increasing trend in T_w_ occurred from 1991 to 2015 (Fig 7), which can be explained by increasing T_a_ (Table 1; Fig 8). Similar shifts in the T_w_ regime in the late 1980s and early 1990s were previously detected in numerous European surface waters, e.g. in Germany [65], Sweden [66] and Switzerland [67], and were attributed to global climate change [67].

During the vegetation period, hydrology and biological activity (both affected by climate change) are dominant drivers, affecting P retention and TP concentrations in the epilimnion of stratified water bodies, including SL [23, 39, 68, 69]. Since the early 1990s, we observed that the summer average TP concentrations in SL began to decrease during periods of low Q, but increased when Q was high. No such relationship was detected in 1963–1991 (Fig 4). This phenomenon may be primarily associated with hydrological changes since the 1990s when Q began to fluctuate more, with both the frequency of low Q periods and the magnitude of high Q events increasing. During low Q periods, thermal stratification was stable in the SL and the epilimnetic TP was rapidly utilized by phytoplankton and removed by settling, thus enriching the hypolimnetic TP pools [68, 69]. When a high Q event disrupted the thermal stratification, P-rich hypolimnetic water was mixed into the epilimnion, which increased TP concentrations in this productive zone. At the same time, the water retention time was shortened at high Q, resulting in reduced retention of the inflow P and its short circuiting through the hypolimnia of the Orlík and Slapy reservoirs, so that the TP concentrations in SL increased even more (Fig 5). It can be assumed that these processes proceeded previously to the 1990s as well, but their effect was lower due to more balanced Q, hence not resulting in such large (and Q-dependent) changes in the epilimnetic TP concentrations.

The trend of declining summer TP concentrations during dry periods (Fig 3), resulting from decreased Q and prolongation of thermal stratification, has been observed elsewhere in recent decades [64, 70, 71]. Additionally, the increasing tendency for disruptions to thermal stratification due to storm events, resulting in increased nutrient supply to the epilimnion from deeper water layers, followed by eutrophication, was also detected in other water reservoirs [64, 72].

The high dispersion of the autumn average TP concentrations and the absence of any trends were apparently caused by variable timing of the onset of the water column mixing at the end of summer stratification. The autumn mixing of the SL water column occurred irregularly from the end of August till October, depending on Q and weather conditions during particular years. High Q was usually associated with cold and rainy weather and resulted in early mixing and increased TP concentrations in the SL surface water. In contrast, the epilimnetic TP concentrations remained low in late summer and early autumn under conditions of low Q and mild weather, which prolonged the duration of thermal stratification.

While the shifts in socio-economic development and climate conditions occurred mainly in 1990–1991, the breaking point of TP concentrations was found in 1992. This delay in the response of water chemistry to the detected changes in P sources can be explained by water residence time in SL and the upstream reservoirs, which can be up to one year [45].

## Conclusions

Analysis of long-term data on P concentrations in SL showed that variations in the epilimnetic TP concentrations had a clear seasonality and were associated with different stages of socio-economic development and climate change drivers. The increasing and decreasing trends in TP concentrations during 1963–1991 and 1992–2015, respectively, were detected in winter and spring. These trends were mainly driven by changes in anthropogenic activities, such as P loads from sanitary systems and agriculture. Changes in the Czech economy and environmental legislation obviously played significant roles in the reduction of P loads in 1992–2015. The summer patterns of TP concentrations were more complex and primarily related to changes in climate and hydrology that become apparent after 1991 (rising water temperature and increasing frequency of low and extreme flows). Low Q supported longer periods of stratification and low epilimnetic TP concentrations. In contrast, high Q events caused disruptions of thermal stratification, mixing of deep (P-enriched) water layers with the epilimnion, and increased epilimnetic TP concentrations. Hence, the variability of summer TP concentrations has increased and begun to be Q-dependent in SL since the early 1990s. This mechanism results in a paradoxical situation when the summer epilimnetic TP concentrations may increase at high Q and the SL epilimnion may become more eutrophic than in the past, despite the general decreases in the external P loads and winter and spring average in-lake TP concentrations.

Our results demonstrate that climate change may lead to a greater susceptibility of aquatic ecosystems to the supply of nutrients and results in elevated eutrophication even at stable or decreasing external P loads. This conclusion highlights the necessity of further reductions of external P sources. Therefore, water managers and policymakers should continue in their efforts to eliminate P pollution in catchments, because the confounding effects of climate change may cause the achievement of the necessary ecological quality standards under the EU Water Framework Directive to become impossible without an additional reduction of P loads to lakes.
